# Mother-to-Infant Bonding in Women with Postpartum Psychosis and Severe Postpartum Depression: A Clinical Cohort Study

**DOI:** 10.3390/jcm9072291

**Published:** 2020-07-19

**Authors:** Janneke Gilden, Nina M. Molenaar, Anne K. Smit, Witte J. G. Hoogendijk, Anna-Sophie Rommel, Astrid M. Kamperman, Veerle Bergink

**Affiliations:** 1 Department of Psychiatry, Erasmus University Medical Center, 3015 GD Rotterdam, The Netherlands; nina.molenaar@mssm.edu (N.M.M.); aksmit92@gmail.com (A.K.S.); w.hoogendijk@erasmusmc.nl (W.J.G.H.); a.kamperman@erasmusmc.nl (A.M.K.); veerle.bergink@mssm.edu (V.B.); 2 Department of Psychiatry, Icahn School of Medicine at Mount Sinai, New York, NY 10029, USA; anna.rommel@mssm.edu; 3 Epidemiological and Social Psychiatric Research Institute, Erasmus University Medical Center, 3015 GD Rotterdam, The Netherlands; 4 Department of Obstetrics, Gynecology and Reproductive Science, Icahn School of Medicine at Mount Sinai, New York, NY 10001, USA

**Keywords:** postpartum depression, postpartum psychosis, mother-to-infant bonding, attachment, mother baby unit, mania, postnatal

## Abstract

Mother-to-infant bonding is important for long-term child development. The aim of this study was to investigate bonding in women admitted to a Mother and Baby Unit with postpartum depression (PD, *n* = 64) and postpartum psychosis (PP, *n* = 91). Participants completed the Postpartum Bonding Questionnaire (PBQ), the Edinburgh Postnatal Depression Scale (EPDS) and the Young Mania Rating Scale (YMRS) weekly during admission. At admission, 57.1% of women with PD had impaired bonding, compared to only 17.6% of women with PP (*p*-value < 0.001). At discharge, only 18.2% of women with PD and 5.9% of women with PP still experienced impaired bonding (*p*-value = 0.02). There was a strong association between decrease of depressive and manic symptoms and improved bonding over an eight-week admission period. In a small group of women (5.7%) impaired bonding persisted despite being in remission of their psychiatric disorder. The results from our study show that impaired bonding is a more present and evidently severe problem in postpartum depression but not so much in postpartum psychosis. Treatment of depressive symptoms will improve bonding in almost all women, but clinicians should assess if impaired bonding is still present after remission because for a small group special care and treatment focused on bonding might be required.

## 1. Introduction

Affective and protective feelings towards the child usually begin during the first trimester of pregnancy [1]. These feelings gradually increase during pregnancy, particularly in response to fetal movements [2]. After giving birth, the term “bonding” refers to the unidirectional feelings experienced by a mother towards her child [3]. Mother-to-infant bonding is considered to form the foundation for the child’s later attachment [4]. The development of bonding is important, as impaired bonding can impact the child’s emotional, cognitive, and behavioral long-term development. For example, studies have shown associations between impaired mother-to-infant bonding and child behavior problems in early childhood [5], and an increased child’s risk of developing psychopathology in adulthood [6]. Moreover, mothers who reported impaired mother-to-infant bonding experienced high levels of parenting stress during toddlerhood [7], and these children are at risk for maltreatment later in life [8]. While in most situations adequate bonding develops gradually after birth, bonding can be impaired when the mother suffers from a psychiatric disorder [8].

The postpartum period is considered a time of increased risk for the development of severe psychiatric disorders [9]. Postpartum depression (PD) is one of the most common disorders and affects approximately 10% of new mothers [10]. A less common but more severe postpartum illness is postpartum psychosis (PP). Postpartum psychosis is the umbrella term for postpartum mania, psychosis, and depression with psychotic features [11]. First onset postpartum psychosis has an incidence ranging from 0.24 to 0.6 women per 1000 births [12,13,14]. The prevalence of PP in women with a prior diagnosis of bipolar disorder is high, our meta-analysis reported 17% [15]. Given the severity of the illness and the high relative risk for suicide and infanticide, early recognition and adequate treatment is of great importance [16,17]. Both severe PD and PP are indications for psychiatric hospitalization [12,18]. Joint inpatient mother–baby admission to a Mother and Baby Unit (MBU) is preferred for PP and PD, as these admissions are associated with reduced time to recovery, when opposed to mother-only admission [19,20,21]. With an adequate treatment regime, nearly all women with postpartum mood disorders achieve full remission [22].

While the association between PD and impaired bonding is well established in current literature [3,23,24,25,26,27], there is hardly any research on the association between PP and impaired bonding. As the underlying nature of PD and PP differ substantially, it is uncertain whether the magnitude of the problem is comparable in both groups. In addition, previous studies have not investigated how the relationship between PD/PP and impaired bonding is affected by the treatment of the depressive and manic symptoms. Acquiring this data could provide insights into driving factors behind impaired bonding and underline the importance of particular treatment regimens. In this inpatient study on a Mother and Baby Unit (MBU), we therefore examined the differences in mother-to-infant bonding between women with postpartum psychosis and women with severe postpartum depression. Additionally, we investigated the association between change in depressive and manic symptoms and mother-to-infant bonding scores over the course of MBU admission.

## 2. Methods

### 2.1. Study Design and Participants

The present study was embedded in an ongoing prospective multicenter cohort study (OPPER), which focuses on the prevention, treatment, and neurobiology of peripartum mood disorders [22,28]. Women are eligible for this study if they (a) are between 18–45 years of age, (b) have a postpartum onset of psychosis, mania, or severe depression as assessed with the Structured Clinical Interview for DSM-IV Axis I disorders (SCID) [29], and (c) are admitted to the MBU. Exclusion criteria are: (a) prenatal onset of psychosis, mania or severe depression, (b) drug/alcohol dependence in the last 3 months, (c), intellectual disability (IQ < 80), (d) serious somatic illness and (e) inability to read or write. The Medical Ethical Committee of the Erasmus Medical Center Rotterdam approved the study (MEC-2005226). Written informed consent was obtained from all participants.

For the current study, all women who participated in OPPER between May 2005 and January 2013, and who had completed a Postpartum Bonding Questionnaire (PBQ) [30], were included.

### 2.2. Data Collection and Procedures

Psychiatric diagnosis was established at intake using the SCID [29]. Bonding was measured with the Postpartum Bonding Questionnaire (PBQ) [30]. The PBQ is a self-report instrument that consists of 25 statements that are rated on a 6-point Likert scale (from “always” to “never”). It has four scales representing (1) impaired bonding, (2) rejection and anger, (3) anxiety about care, and (4) risk of abuse. The maximum total score is 125 with a cut-off of 26 to identify mothers with medium impaired bonding and a cut-off of 40 for mothers with severe impaired bonding. Depressive symptoms were measured with the widely-used Edinburgh Postnatal Depression Scale (EPDS) [31]. A score of 13 or higher is indicative of depressive disorder [31,32]. Manic symptoms were measured using the Young Mania Rating Scale (YMRS) [33], a well-validated 11-item interviewer rated scale designed to assess the severity of manic symptoms in bipolar patients. A state of euthymia usually requires a score below the cut-off of 12 [34]. During hospitalization, the PBQ and EPDS were completed weekly by the participant and the YMRS was completed weekly by a trained rater.

### 2.3. Treatment

Women in this study received pharmaceutical treatment according to medical guidelines, as described previously [35]. Women with PP were treated with antipsychotics and/or lithium, while women with PD were treated with either tricyclic antidepressants or a selective serotonin reuptake inhibitor. In addition, non-pharmacological interventions to optimize mother–baby interaction took place as part of the regular program of the MBU. These interventions included guidance from nursing staff, video interaction guidance, baby massage and a support group for mothers. Women with PD and PP were equally exposed to these interventions. Information on the effect of each individual treatment intervention on mother-to-infant bonding was not collected in this observational study.

### 2.4. Statistical Analysis

Descriptive statistics of the study population are provided. Differences in demographic and clinical characteristics between the PD and the PP group were tested using *t*-tests, Mann-Whitney U tests, chi-square tests and Fisher’s exact test. We then tested differences in PBQ scores (both dichotomized using the cut-off score of 26 and the cut-off score of 40) between women with PD and women with PP at admission and at discharge, using chi-square tests and Fisher’s exact test. Additionally, we tested whether bonding scores showed a different course during the MBU admission period for PD and PP women by testing a diagnosis*time interaction term using linear mixed effect modelling analysis (LMM, fixed intercept, fixed slope, AR(1), covariance matrix, maximum likelihood estimation). To examine the association between change in depressive and manic symptoms and bonding scores over the course of MBU admission, we used linear mixed effects modelling as well. This model ensures optimal use of the weekly repeated assessments over the course of admission [36]. Because depressive symptoms were present in both the PP and the PD groups, we tested the association between depressive symptoms and bonding in both groups separately. Manic symptoms are, by definition, exclusively present in the PP group. We therefore tested the association between manic symptoms and bonding in the PP group only. Analyses were adjusted for maternal age and primiparity [16,37,38,39]. We report coefficients and their confidence intervals. To visualize these associations, we created graphs displaying mean EPDS, YMRS, and PBQ scores over the first eight weeks of admission in both the PD and the PP group. We chose to display eight weeks as this was the mean length of admission. Statistical Package for Social Sciences (SPSS) version 24.0 was used for data analysis. All hypotheses were tested with an alpha of 0.05 (two-sided).

## 3. Results

Participants were 155 women with a severe postpartum mood disorder, of which 91 were diagnosed with postpartum psychosis and 64 were diagnosed with postpartum depression. Relevant demographic and clinical characteristics are presented in Table 1. Infant age at admission was significantly higher for women with PD compared to women with PP. As expected, depressive symptoms (EPDS) were higher in women with PD. There were no substantial differences in maternal age, length of admission, relationship status, education level, gender of the infant and psychiatric history of the women.

### 3.1. Prevalence of Impaired Mother-to-Infant Bonding at Admission and Discharge

**Figure 1:**
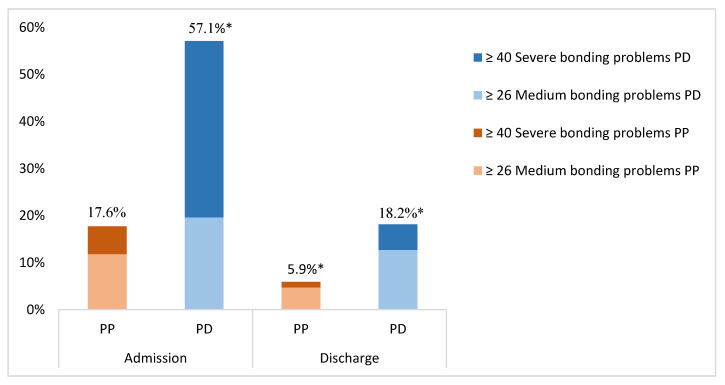
Proportion of women with PP and PD presenting with medium to severe impaired bonding at hospital admission and discharge; * = Significant result (*p* < 0.05); Abbreviations: PP = postpartum psychosis; PD = postpartum depression; Note: PBQ scores were missing at random at both time-points. During admission PP (*n* = 68)/PD (*n* = 56), at discharge PP (*n* = 85)/PD (*n* = 55).

At admission, 17.6% of women with PP compared to 57.1% of women with PD had a PBQ score above the cut-off of 26 (*p*-value < 0.001), which is associated with medium to severe impaired bonding (Figure 1). At discharge, only 5.9% of women with PP compared to 18.2% of women with PD still had a PBQ score above this cut-off (*p*-value = 0.02).

**Figure 2:**
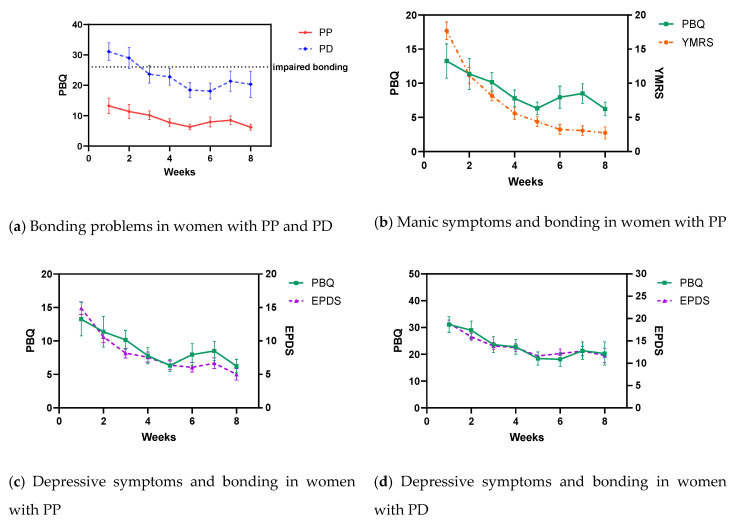
Change in mean mother-to-infant bonding scores and depressive (EPDS) and manic (YMRS) symptoms in women with postpartum psychosis and postpartum depression (including standard error bars). Abbreviations: PP = postpartum psychosis; PD = postpartum depression; PBQ: Postpartum Bonding Questionnaire; YMRS = Young Mania Rating Scale; EPDS = Edinburgh Postnatal Depression Scale.

The percentage of women with severe impaired bonding (PBQ score above the cut-off of 40) at admission in women with PP was 5.9% compared to 37.5% in women with PD (*p*-value < 0.001). At discharge, these percentages decreased to 1.2% in women with PP compared to 5.5% in women with PD (*p*-value = 0.30). Mean PBQ scores during the first eight weeks of admission per group can be seen in Figure 2. The PBQ scores went down gradually during the first five weeks of MBU admission for both PP and PD patients, but showed no further decline up to eight weeks of admission. Formal testing showed that the course of bonding problems during the admission period differed significantly between PP and PD women (F(672) = 8.632; *p* = 0.003).

### 3.2. Persistent Impaired Bonding at Discharge

At discharge, 15 women in total (10.7%) continued to experience impaired bonding (5 women with PP and 10 women with PD). In seven of these women (5.0%), depressive symptoms (EPDS > 12) were also still present. In eight women (5.7%), impaired bonding remained present in the absence of depressive symptoms. None of these women had manic symptoms.

### 3.3. Postpartum Depressive and Manic Symptoms and the Association with Mother-to-Infant Bonding

Depressive symptoms decreased gradually over the course of admission. At admission, the mean EPDS score in the PD group was 19.0 (SD 4.9) and 14.9 (SD 6.0) in the PP group, which decreased to 10.3 (SD 6.0) and 4.1 (SD 4.6) respectively. In the PP group, manic symptoms decreased gradually over the course of admission as well, from a mean YMRS of 17.7 (SD 11.5) at admission to a mean YMRS of 1.1 (SD 1.6) at discharge. Table 2 demonstrates the results of the association of maternal depressive (EPDS) and manic (YMRS) symptoms and mother-to-infant bonding (PBQ) during inpatient follow-up, based on linear mixed effects modelling. There was a significant association between a decrease in depressive symptoms and improvement of bonding over the 8-week admission period in PD patients (Figure 2d). A similar, but slightly weaker association between a decrease in depressive symptoms and improvement of bonding was seen in the PP group (Figure 2c). In addition, we found a significant association between a decrease in manic symptoms and improvement of bonding in PP patients (Figure 2b), independent from the association with depressive symptoms. However, the correlation between depressive symptoms and impaired mother-to-infant bonding was much stronger (compared to the correlation between manic symptoms and impaired mother-to-infant bonding).

## 4. Discussion

### 4.1. Differences in Bonding Problems between PP and PD

In this prospective cohort study, we found that impaired mother-to-infant bonding is a major problem in inpatient women with severe postpartum depression (57.1%), while less than 1 out 5 women with postpartum psychosis reported impaired bonding (17.6%).

Moreover, in women with postpartum depression we found a higher proportion with severe impaired bonding compared to women with PP. This is in line with previous research, which reports that depressed mothers perceived their bonding to the baby more negatively than psychotic mothers during admission [40]. In this study, they observed impaired bonding rates in 61.1% of women with PD and 29.4% of women with PP. We agree with Hornstein et al. [40] that this intergroup difference is an expression of the nature of the psychopathology. Mothers with PD have feelings of inadequacy, negative cognitions, and self-doubt resulting in a negative bonding experience, while most mothers with PP lack these cognitions, especially if they mainly have manic symptomatology.

At discharge, the number of women reporting impaired bonding had greatly reduced in both groups (5.9% in PP vs. 18.2% in PD). Studies on impaired bonding in the general population have shown prevalence rates from 12.2% at 48 hours postpartum [41] to 7.1% and 8.9% at 2 and 12 weeks postpartum respectively [42,43]. Thus, the prevalence rate of impaired bonding at discharge in women with a diagnosis of PP could be considered equal to that of the general population.

### 4.2. The Effect of Treating Depressive and Manic Symptoms on Mother-to-Infant Bonding

A recent systematic review identified 29 previous cross-sectional studies that investigated the correlation between depressive symptoms and postnatal mother-to-infant bonding [27]. Out of these 29 studies, 21 studies observed a moderate to strong correlation (r = −0.61 to −0.14, ß = −0.39 to −0.26). None of these studies examined the correlation over time. In our longitudinal study, we observed the correlation between depressive symptoms and mother-to-infant bonding over time, and found an almost exact concordant movement of both scores over an 8-week admission period. This indicates that improvement in mother-to-infant bonding is highly dependent on improvement of depressive symptoms. Impaired bonding seems to be an integral part of postpartum depression and could therefore be an indication of the presence of a postpartum depression. We also found a correlation between manic symptoms and mother-to-infant bonding over time, although this correlation was much weaker.

In a small group of women (5.7%), impaired bonding persisted despite effective treatment of the depressive and/or manic symptoms. This finding is in agreement with the earlier observations by other researchers [3,8,44], and with reported prevalence rates of impaired bonding in the general population, which suggest that impaired bonding can exist independent of psychiatric symptoms. Therefore, at discharge, and also when patients are in remission, the quality of the mother-to-infant bonding should be assessed. For these women, besides treatment of their psychiatric disorder, interventions aimed at improving bonding are important.

### 4.3. Strengths and Limitations

Although the findings should be interpreted with caution, this study has several strengths. This is the first prospective cohort study to investigate the association of longitudinal depressive and manic symptoms (at the symptom level) with mother-to-infant bonding in patients admitted with postpartum psychosis or severe postpartum depression. Another strength of this study is the use of the PBQ [30], an extensive screening instrument, assessing all domains of bonding. Nonetheless, the current study also has several limitations. Differentiation between the most effective interventions for impaired bonding could not be established, because inpatient treatment at the MBU included both interventions aimed at reducing depressive, manic and/or psychotic symptoms, and interventions focused on optimizing the mother–baby interaction. However, our positive results for improvement on both domains underline the added value of joint inpatient mother–baby admission to an MBU as opposed to mother-only admission. Furthermore, we encountered missing data points for measurements during admission. Fortunately, mixed models allow data for all subjects to be included in the analysis regardless of whether they completed all study time points.

### 4.4. Clinical Implications

The results from our study implicate that women with severe postpartum psychiatric disorders, and especially women with severe postpartum depression, are at risk of impaired mother-to-infant bonding due to the nature of their symptoms. While both manic and depressive symptoms were strongly associated with impaired bonding, the association with depressive symptoms was stronger both in the PD and the PP groups. Over the course of eight weeks of inpatient mother–baby admission at our Mother and Baby Unit (MBU), the combined pharmacological and non-pharmacological treatment greatly improved both depressive and manic symptoms and mother-to-infant bonding. While these results are encouraging, admission to an MBU is only reserved for mothers with severe postpartum psychiatric disorders and contingent on the presence of an MBU. Many women with mild to moderate symptoms of depression will go undetected and untreated, or will receive treatment untailored to the postpartum period, posing a threat to adequate mother-to-infant bonding, putting the child at risk for adverse short- and long-term outcomes. Our findings support the necessity to establish MBUs, or at least tailored treatment programs, for optimal patient care. Since the prevalence of prenatal depression is similar or even higher than postpartum depression [45,46,47], and mother-to-infant bonding already starts in the first trimester of pregnancy, we strongly encourage that diagnosis and interventions should take place as early as possible during the perinatal period to avoid harmful consequences. Screening for depression during pregnancy and the postpartum period is unfortunately not yet standard clinical practice in many settings. Interventions should primarily be focused on the treatment of the psychiatric disorder. Nonetheless, after remission has been achieved, it is important to check for residual impaired bonding and to act accordingly.

### 4.5. Implications for Future Research

The results from our study indicate that mother-to-infant bonding is a major problem in women with postpartum psychiatric disorders, especially in women with severe PD. Our data suggests that treatment of depressive symptoms improves mother-to-infant bonding in almost all women. There is an urgent need for studies comparing improvement in psychiatric symptomatology and mother-to-infant bonding in MBU settings compared to regular outpatient treatment settings. In addition, longer follow-up studies in women with impaired mother-to-infant bonding should be initiated and include measurements of the cognitive and emotional development of the child, to investigate the long-term effect of impaired bonding on the offspring later in life.

## Figures and Tables

**Table 1:** Demographic and clinical characteristics.

	Postpartum Psychosis (*n* = 91)	Postpartum Depression (*n* = 64)	*p*-Value
Maternal age at admission (yr), mean (SD)	31.0 (4.7)	30.0 (8.0)	0.32
Length of admission (wks), mean (SD)	7.9 (4.3)	7.5 (4.8)	0.52
Dutch nationality, *n* (%)	83 (92.2)	55 (90.2)	0.66
Relationship, yes, *n* (%)	88 (97.8)	55 (93.2)	0.21
Primary/Secondary education only, *n* (%)	44 (48.4)	33 (60.0)	0.17
Primiparous, *n* (%)	68 (78.2)	40 (64.5)	0.09
Psychiatric history, *n* (%)			
None	44 (48.4)	25 (39.1)	0.14
Prior postpartum episode	11 (12.1)	4 (26.7)	
Prior non-postpartum episode	36 (39.6)	35 (54.7)	
EPDS score at admission, mean (SD)	14.9 (6.0)	19.0 (4.9)	0.001 *
YMRS score at admission, mean (SD)	17.7 (11.5)	n.a.	n.a.
Family history of psychiatric disorder, *n* (%)	51 (56.0)	20 (35.0)	0.013 *
Infant age at admission (wks), median (IQR)	2.0 (3.7)	6.0 (5.2)	<0.001 *
Gender infant, male, *n* (%)	45 (50.0)	26 (43.3)	0.51

* = Significant result (*p* < 0.05).

**Table 2:** The effect of depressive (EPDS) and manic (YMRS) symptoms on mother-to-infant bonding (PBQ).

Fixed Effects	Unadjusted Estimate	Sig.	Adjusted Estimate	Sig.
Linear mixed effects model in PP group				
Intercept	2.62	0.02	7.96	0.22
EPDS	0.76	0.00	0.76	0.00
YMRS	0.16	0.02	0.16	0.02
Linear mixed effects model in PD group				
Intercept	8.30	0.00	1.98	0.88
EPDS	1.05	0.00	1.05	0.00

Abbreviations: PP = postpartum psychosis; PD = postpartum depression; EPDS = Edinburgh Postnatal Depression Scale; YMRS = Young Mania Rating Scale; PBQ = Postpartum Bonding Questionnaire. Adjusted estimates: adjusted for maternal age and primiparity.
